# Providers and patients face-to-face: what is the time?

**DOI:** 10.1186/s13584-017-0180-1

**Published:** 2017-10-10

**Authors:** Andrew D. Racine

**Affiliations:** 1 Montefiore Health System, Bronx, New York USA; 2 Albert Einstein College of Medicine, Bronx, New York, USA

## Abstract

**Background:**

The frequency of visiting primary care providers and the duration of those visits varies substantially by patient demographics and across different developed countries. The significance of a cumulative measure of this time spent with providers in face-to-face visits is not well understood.

**Commentary:**

In a recent *IJHPR* issue Nathan and co-authors have suggested a new metric for capturing the cumulative time spent annually in face-to-face encounters between providers and patients. The annual accumulated duration of time (AADC) of visits was constructed using a 2% random sample of adult patients from the Clalit health plan in Israel for the year 2012. The authors calculated the mean AADC to be 65.7 min with average visit durations of 7.6 min. A presumption underlying this analysis is that the metric captures the magnitude of activity devoted to eliciting relevant clinical information, synthesizing the significance of those data, and communicating the importance of that thinking to patients so that they might make informed decisions regarding their health care. But measuring the time spent with a provider is but a surrogate marker of these activities and the lack of correlation between time spent with providers and health outcomes suggests that as a surrogate it may not be that robust a measure. It is possible that what is being captured through this metric is the influence of economic incentives faced by individual practitioners and the structure of health care financing in different societies rather than a portrait either of clinical complexity or quality of care.

**Conclusions:**

The advent of this new measure of cumulative provider time with patients signals the importance of accurate measurement as a vital first step in understanding the meaning of data but reminds us of an obligation to inquire beyond the measurements themselves to arrive at appropriate policy-relevant conclusions.

Nathan et al. [1] have given us an interesting metric to measure primary care utilization: the annual accumulated duration of time of visits (AADT). This measure attempts to combine the frequency of primary care visits with their duration to calculate the total face-to-face time that patients experience with providers. Using a random 2% sample of all patients over 18 years of age who received their care from Clalit Health Services (*n* = 77,247), the largest health maintenance organization in Israel, the authors found that in 2012 the average number of visits with a PCP was 8.8 (+/− 9.1) while the average total amount of time with the PCP was 65.7 (+/− 75.8) minutes so that the average duration of a single visit was 7.6 (+/− 4.3) minutes. More total annual time was recorded for women, for older patients, for those with more chronic conditions, those from lower socio-economic backgrounds, and for those living in kibbutzim (relative to city-dwellers). Immigrants, by contrast, had fewer total face-to-face minutes with their providers over the course of a year. The authors speculated initially that from the standpoint of adjusting capitated payments to health plans in Israel it might make more sense to use the annual accumulated duration time metric rather than simply visit numbers, but their analysis revealed that visit duration was little altered by age and gender. Consequently visit number was closely correlated to total annual time spent with the provider so that it functioned fairly well as a proxy for the AADT.

If, as the authors postulate, AADT captures more completely than visit number alone the attention that patients are receiving from their providers over the course of a year, the salient question raised by this study, and others like it, is why should we care to know this. If it is to guide allocation of resources either to payers or providers as one form of risk adjustment one must ask whether, once health status and diagnostic complexity is accounted for, would one run the risk of rewarding inefficiency to direct more resources to providers who, these other features held constant, are taking greater time to treat their patients. If, on the other hand, time spent with patients is itself a marker of quality then it might certainly make sense to fashion a payment schedule that encourages, to a point, greater time with patients at any given level of disease complexity. To answer these questions we must first arrive at some understanding of what exactly is being measured here.

Fifty years ago in the *American Economic Review*, William Baumol called our attention to the distinction between economic activity in which labor is primarily an instrument, “requisite for the attainment of the final product” and other fields of economic activity in which labor is itself the end product. [2] Throughout many succeeding years Baumol and his colleagues clarified that in the medium to long term productivity increases that take place in the former type of activity far outstrip those in the latter. When manufacturing an automobile, or a dishwasher, or a computer the processes of production can over time infuse greater and greater amounts of capital and technology such that fewer and fewer person hours are necessary to produce the same output. That is not true of those areas that contain an irreducible quantity of human labor beneath which the activity ceases to function properly. [3] As Baumol astutely pointed out:

A half hour horn quintet calls for the expenditure of 2 ½ man hours in its performance, and any attempt to increase productivity here is likely to be viewed with concern by critics and audience alike [1].

Clinical interactions that occur between providers and patients surely fall into this second type of category. To interview a patient, take a careful history, perform a thorough physical examination, and explain to the patient diagnostic conclusions and the potential therapeutic alternatives – all this takes time. As patients age and the complexity of their conditions increases, this requisite time also increases *pari passu*. As primary care becomes the venue in which a host of social problems must also be screened for, identified, and addressed, the time necessary to accomplish this important set of tasks expands concomitantly. What primary care practitioners the world over are struggling with is how best to accommodate these seemingly inexhaustible claims on patient-provider time together. One can make marginal changes through the judicious use of technology: having patients fill out important information before the visit, using electronic medical records to decrease the time necessary to locate pertinent information that can inform the discussion between patient and provider, recruit novel technologies to provide face-to-face encounters through other platforms than direct visit time, etc. But these technological applications do not, in the end, entirely replace or necessarily even diminish, the face-to-face time that sits resolutely and immovably at the center of clinical medicine. That being the case, what does this time spent represent and is it worth measuring? If time spent with the provider is a measure of health care service delivery quality then it would make good sense to want to know the magnitude and trends associated with this metric. If that is not the case, measuring AADT or any other marker of time spent with the provider may be of less consequence.

What we are attempting to measure with visit numbers, visit duration, or AADT is that portion of health preserving or health enhancing activity that is derived from periodic encounters between the primary care practitioner and the patient. This activity involves the eliciting of information about the patient’s condition, the synthesis of that information to construct a coherent understanding of the patient’s health trajectory, the communication of that understanding to the patient, and the evaluation of what interventions are most sensible to the patient based on his or her understanding of the accumulated information and appetite for risk, inconvenience, willingness to engage and a host of other characteristics.

But time itself is but a surrogate marker for these activities. It is not known the extent to which any or all of this activity occurs in a given encounter or even across a series of multiple encounters. Although we have some indication that at least in an American context longer duration of visits may be associated with increased screening in certain instances, [4] and international comparisons have shown an association between specific characteristics of primary care and mortality rates, these same analyses using multivariate models showed no association between the *number* of visits per capita and either mortality rates or potential years of life lost. [5]

Nor are these the only observations suggesting that time with the provider and health status may not be very highly correlated. Consider the differences between Israeli and American estimates of time with providers that Nathan et al. have illuminated. While the Israelis surveyed in Nathan et al.’s study spent an average of 65.7 h a year with their providers, Americans were together with their providers far less. The latest American estimates are that in 2014 the average number of visits to primary care providers was 1.47 in the U.S. and the median time spent with the physician was between 17.8 and 19.3 min. [6] This would roughly translate into an AADT of between 26.2 and 28.4 min, or a little more than 40% of the time devoted in Israel. While U.S. health outcomes are by many measures less optimal than those achieved in Israel, they are not as inferior as those proportions of time would indicate.

So if time with the provider is not a good measure of health care quality (at least as captured in health outcomes) what is it an indicator of? It may well be that when we measure the time spent between provider and patient what we are actually capturing is a reflection not of quality of care being delivered but rather the underlying economic incentives faced by the primary care practitioner. In the U.S., where the majority of care is still delivered in a fee-for-service environment, spending increasing amounts of scarce time on the same patient does not return the same amount to the provider as using that time to see a new patient where the reimbursement is generally higher. Only in a capitated environment in which one is accountable for the health outcome of the patients assigned to a provider are the economic incentives more comparable to what is faced in the Israeli context. Furthermore, in the U.S. time with the provider is considerably more expensive than time with the provider in Israel given the disparity of incomes between providers in the two countries. In 2003 OECD statistics listed the mean annual income of general practitioners in the U.S. to be $146,000 or 3.4 times the average wage. [7] In 2011 the comparable figure for Israel was $66,000 in USD [8] or about twice the average wage. [9] That being the case, as a factor of production it makes sense to conserve physician time for any given patient in the former context relative to the latter. If time with the provider is relatively inexpensive, spending more of it to achieve a desired health outcome makes good sense.

## Conclusions

Nathan et al. have done a considerable service with their analysis of how we might measure more comprehensively the time that providers spend with their patients. The challenge now is to dissect the meaning of this or any measure of time with patients in order to advance the policy debate over the use of scarce health care resources. When we do this, we may find ourselves confronting the inescapable irony that the scrutiny we devote to the object of our interest obscures as much as it reveals.

## Abbreviations

AADC: Annual accumulated duration of time of visits
